# Solitary Giant Simple Hepatic Cyst

**DOI:** 10.14309/crj.0000000000001170

**Published:** 2023-10-03

**Authors:** Victoria Diaz, George Trad, Shruthi Narasimha, Syed A. Basit

**Affiliations:** 1 Department of Internal Medicine, Sunrise Health Graduate Medical Education Consortium, Las Vegas, NV; 2 Gastroenterology Fellowship, Sunrise Health Graduate Medical Education Consortium, Las Vegas, NV

## CASE REPORT

**Figure 1.**
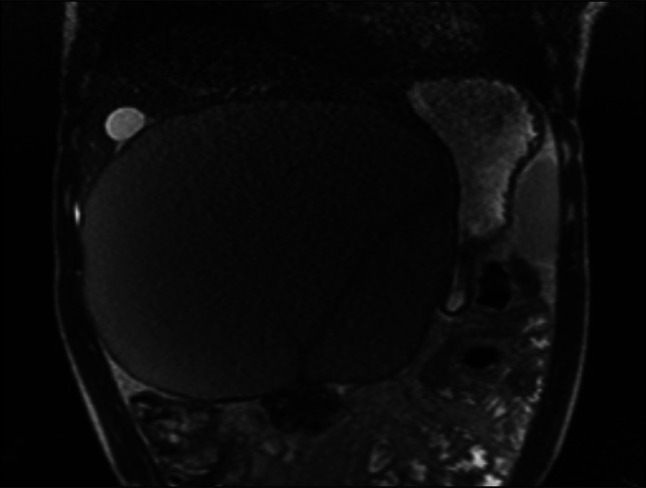
Magnetic resonance imaging of the abdomen with intravenous contrast in coronal and axial view showing a large subhepatic cystic mass, measuring 23.0 × 16.0 × 21.0 cm, which abuts the liver along its superior border.

**Figure 2.**
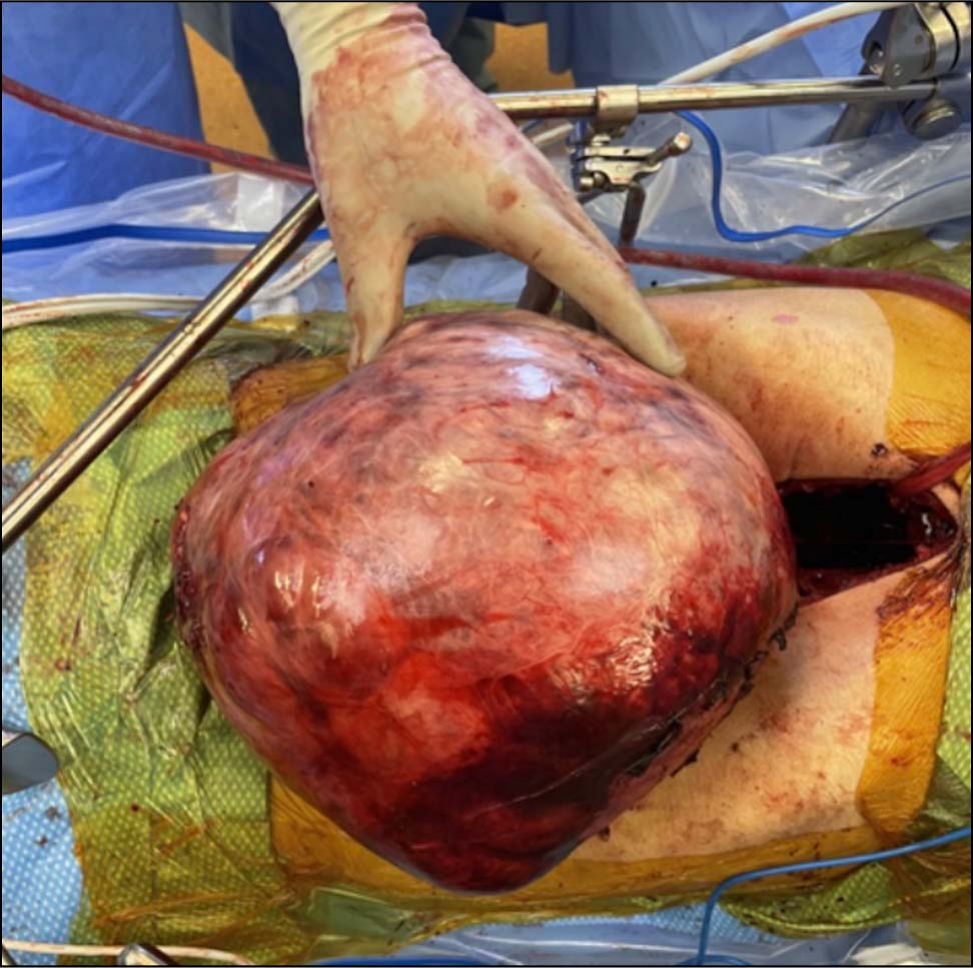
Gross surgical specimen image of the hepatic cyst. The cyst drained 1,200 mL of crystalloid fluid. Gross description described the specimen as weighing 4,459.5 g, with the cystic portion of tissue variegated tan to red-brown with a smooth to focally trabeculated external surface.

An 18-year-old healthy man presented to the emergency department with complaints of a 2-week history of right upper quadrant abdominal pain. Vitals were stable on presentation. On physical examination, the abdomen was soft with right upper quadrant prominence and mild tenderness. Initial laboratory studies showed no leukocytosis, low normal platelets at 169 (10^3^/μL), and normal total bilirubin, but aspartate aminotransferase, alanine aminotransferase, and alkaline phosphatase were 176, 361, and 221 (U/L), respectively. Hepatitis panel, HIV, and echinococcus testing were negative. Magnetic resonance imaging with and without intravenous contrast showed large subhepatic cystic mass, measuring 23.0 × 16.0 × 21.0 cm (Figure 1) with compression of the main portal vein leading to mild splenomegaly and varices. Owing to pain, the patient underwent an exploratory laparotomy with partial hepatectomy of the caudate lobe by the oncological surgery team for enucleation of the cyst via intraoperative ultrasound-guided dissection (Figure 2). Sectioning demonstrated a multiloculated cystic cut surface with hemosiderin-laden macrophages and no atypia or malignancy. Hepatic cysts are often common incidental findings with a prevalence that ranges from 2.5% to 18%.^1^ Our case is the third largest liver cyst in literature and is also the only one to show portal hypertension as a complication.^2–5^

## DISCLOSURES

Author contributions: All authors contributed to the report by obtaining case details, outlining summary, and approving the final document. We are all responsible for the details in this report and agree to be accountable for any enquires in our summary. S. Narasimha is the article guarantor.

Financial disclosure: None to report.

Informed consent was obtained for this case report.
